
JOURNAL INFORMATION
==============================
NLM Title Abbreviation: Inter Econ
Journal ID: Inter Econ
NLM Journal ID: 101086399

Inter Economics

ISSN: 0020-5346

ARTICLE INFORMATION
==============================
PMCID: PMC7584854
DOI: 10.1007/s10272-020-0919-8
Article ID: 919
Article version: 1
Subjects: Forum

Reflections on the Stability and Growth Pact’s Preventive Arm in Light of the COVID-19 Crisis

Hauptmeier Sebastian sebastian.hauptmeier@ecb.europa.eu

Leiner-Killinger Nadine Nadine.Leiner-Killinger@ecb.europa.eu

grid.466644.2 0000 0004 0555 7916 Fiscal Policies Division - Directorate General Economics, European Central Bank, Sonnemannstrasse 20, 60314 Frankfurt a.M., Germany
Electronic publication date: 2020 Oct 24
Print publication date: 2020
Volume: 55
Issue: 5
First page: 296
Last page: 300
Copyright: © The Author(s) 2020
License: Open Access: This article is distributed under the terms of the Creative Commons Attribution 4.0 International License (https://creativecommons.org/licenses/by/4.0/).
Open Access funding provided by ZBW — Leibniz Information Centre for Economics.
License URL: https://creativecommons.org/licenses/by/4.0/

issue-copyright-statement: © The Author(s) 2020

==============================
The views expressed in this paper are those of the authors and do not necessarily reflect those of the European Central Bank. The authors would like to thank Joao Semeano Domingues, Bela Szörfi and Vilem Valenta for their input and comments.

Over the last decade and in light of the past great financial crisis, the EU's fiscal framework has been modified in many respects. One major modification with material consequences for the euro area's aggregate fiscal stance pertains to the introduction in 2015 of a so-called matrix of fiscal adjustment requirements under the preventive arm of the Stability and Growth Pact (SGP).[1] For countries that have not achieved their medium-term budgetary objectives, these fiscal adjustment requirements need to feed into their budgetary planning for the next year. Importantly, the matrix has also become the guiding post for adjustment requirements in countries with high government debt ratios, de facto replacing the application of the SGP's debt rule, which had been introduced with the six-pack and two-pack regulations in 2011.

1 On 13 January 2015, the European Commission issued a Communication on "Making the best use of the flexibility within the existing rules of the Stability and Growth Pact", which entered into force with immediate effect.

By the end of 2019, all euro area member states were subject to the SGP's preventive arm. As the devastating COVID-19 shock hit Europe, however, they were allowed to deviate from the fiscal adjustment requirements that would normally apply thereunder, and for the first time since it was introduced in 2011, the SGP's general escape clause was activated. Although deployed independently, this has allowed fiscal and monetary policy to counteract the economic impact of the pandemic in a mutually reinforcing manner. Once the economies have sufficiently recovered, the important fiscal support provided during the crisis will need to be withdrawn and government debt must be reduced.

This article argues that the application of the SGP's preventive arm matrix should cease to be the reference point for such future fiscal adjustment. Among others, the matrix is a reflection of the many inconsistencies in the EU's fiscal framework that have built up over the years. These need to be addressed within the upcoming so-called economic governance review to support a sustained recovery from the deep coronavirus crisis. The shortcomings identified in this article also speak in favour of shifting the EU's fiscal framework towards government debt as an anchor, something that is advocated frequently in the debate on SGP reform. From a central bank perspective, it will be particularly important that a future reform of the fiscal framework internalises the important interactions between monetary and fiscal policies as well as considerations related to the overall macroeconomic policy mix.

## The SGP's preventive arm matrix

As shown in Table 1, the SGP's preventive arm matrix modulates countries' fiscal adjustment requirements for the next year according to the output gap, i.e. the dif-

Table 1
**Matrix specifying the annual fiscal adjustment requirements under the SGP's preventive arm**

| | | Required fiscal adjustment (in percentage points of GDP) | |
|---|---|---|---|
| | **Condition** | Debt < 60% of GDP and no sustainability risk | Debt > 60% of GDP or sustainability risk |
| Exceptionally bad times | Real growth < 0 or OG < -4 | No adjustment needed | |
| Very bad times | -4 ≤ OG < -3 | 0 | 0.25 |
| Bad times | -3 ≤ OG < -1.5 | 0 if growth below potential, 0.25 if above potential | 0.25 if growth below potential, 0.5 if above potential |
| Normal times | -1.5 ≤ OG < 1.5 | 0.5 | > 0.5 [*0.6*] |
| Good times | OG ≥ 1.5 | > 0.5 if growth below potential, ≥ 0.75 if above potential | ≥ 0.75 if growth below potential, ≥ 1 if above potential |

Note: 'OG' refers to output gap.

Source: Stability and Growth Pact, Code of Conduct.

Figure 1
**Revisions to the AMECO output gaps, real time versus *ex post*, 2007-19**

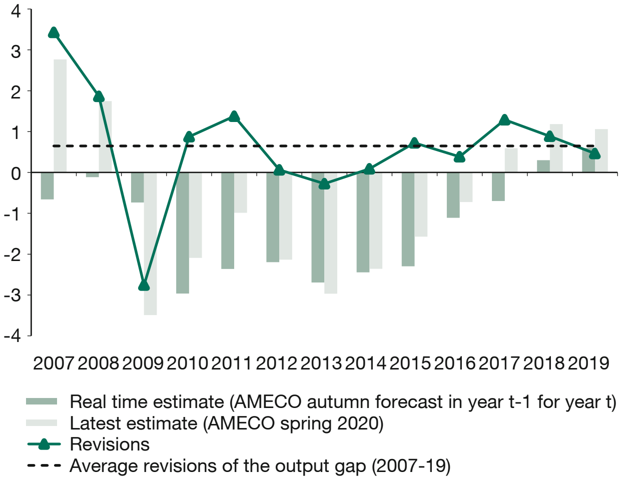

Source: AMECO, own calculations.

ference between real GDP and potential output, and whether the government debt-to-GDP ratio is above or below the Maastricht Treaty's reference value of 60% of GDP. Specifically, the requirements for the budget year t+1 are set in spring of year t. Compliance with these requirements is reviewed in spring t+2 based on outturn data. The partitioning of the business cycle and the output gap as well as the resulting adjustment needs for the structural balance are highly granular. As a result, small decimal-point changes in output gap estimates can be associated with a difference in a country's annual structural adjustment requirement of a quarter percentage point of GDP.

## Problems for policy guidance based on the matrix

### The unobservable output gap

The steering of countries' fiscal stance in the EU's fiscal framework based on the unobservable output gap – the key element of the matrix – is prone to making policy mistakes in real time (Kamps et al., 2014).

Several analyses have shown that in favourable (weak) economic times, unobservable potential output tends to be over(under-)estimated and the output gap under(over-) estimated. As a consequence, in real time, postulated fiscal adjustment requirements risk being smaller (larger) than what the matrix would prescribe for the actual position in the cycle. For the period 2007-19, the output gap as measured by the Economic Policy Committee's commonly agreed methodology[2] (European Commission, 2014) has been underestimated by on average two-thirds of a percentage point (Figure 1).[3] Consequently, with the benefit of hindsight, fiscal adjustment requirements over this period should have been higher on average, notably by way of building higher fiscal buffers in good economic times.[4]

The granularity of the matrix' recommendations for fiscal adjustment and the associated fine-tuning of the fiscal stance based on the unobservable output gap adds a layer of unreliability to the policy prescriptions.[5] To illustrate

2 The Economic Policy Committee's Working Group on Output Gaps has the mandate to improve on the methodology, which is frequently happening, such as the recent introduction of a so-called plausibility tool.
3 When looking only at the period since the inception of the matrix, the average underestimation of the output gap in real time rises to three-quarters of a percentage point. As Figure 1 shows, the forecast error is captured as the difference between the output gap estimate for year t as put forward in autumn t-1 and the *ex post* output gap outturn. Capturing the forecast error as such implies that at least a small part refers to possible errors made in forecasting developments in GDP.
4 Due to a so-called freezing principle, countries are shielded from the need to deliver after the finalisation of the budget year an additional structural adjustment in case the *ex post* estimate of the output gap would have warranted a higher adjustment. In contrast, in case the output gap turns out worse than expected, the adjustment requirement declines *ex post*.
5 The European Commission has shown evidence of such misspecifications of policy advice in real time resulting from the choice of a "wrong" matrix category for a number of years and countries, though it considers that these have not been broad-based across its own forecast vintages. See for details European Commission (2018) on the review of the flexibility under the Stability and Growth Pact, which captured the years 2000-17.

Figure 2

**Output gap estimates: European Commission versus IMF, 2007-21**

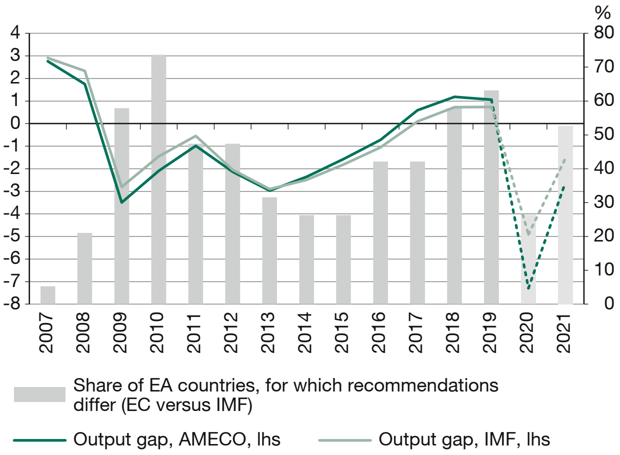

Notes: The bars capture the number of euro area countries in percent of the total, for which the European Commission's and the IMF's forecasts of output gaps fall into a different category under the SGP's preventive arm matrix and would thus tend to imply different structural adjustment requirements.

Sources: European Commission Spring 2020 Economic Forecast and IMF World Economic Outlook, July 2020.

this point, Figure 2 compares the measures of the euro area output gap over 2007-21 as prepared by both the European Commission and the IMF. For the period 2007-19, for which *ex post* data are available, the output gap estimates of both institutions are closely aligned. As *ex post* data on real GDP should be broadly the same for both institutions, differences in *ex post* output gaps should solely reflect differences in potential output estimates. Notwithstanding the closely aligned potential output gap estimates during 2007-19, the fiscal adjustment that both institutions would have prescribed the individual euro area countries to deliver according to the matrix would have differed widely. As the grey bars indicate, if the institutions' policy advice would have been given based on their *ex post* estimates of the output gap, the policy guidance that both institutions would have issued based on the matrix for 2019 would have differed for 60% of the euro area countries. This risks undermining countries' ownership of recommendations for fiscal adjustment under the EU's policy framework.

## Governments' responses to the COVID-19 shock

Governments' responses to the unprecedented shock and the recovery from the coronavirus pandemic make it even more difficult to gauge the unobservable output gap in real time over the medium to long term.

As the COVID-19 crisis hit, European governments enacted economy-wide lockdowns, the impact of which on potential output is very difficult to capture. One extreme interpretation is that the temporary lockdown of an economy does not affect its full production capacity as factors of production remain in place. For example, in most countries, short-time work schemes have allowed workers to maintain ties with their employers. The other extreme interpretation is that a large part of the factors of production have become unavailable during the lockdown, implying a sizeable decline in a country's productive capacity. The choice of interpretations or a mixture thereof has direct consequences for the assessment of an economy's cyclical position.[6]

As Figure 2 indicates, the European Commission and the IMF had very different assessments in mid-2020 of the pandemic's impact on the euro area's potential output.[7] According to the European Commission, potential output is gauged to decline only marginally in 2020, giving rise to a large negative output gap estimate. In contrast, the IMF estimates potential output to decline rather strongly as a result of the pandemic with in a comparably smaller size of the negative output gap. Actually, however, risks of mismeasurement of the output gap during the acute phase of the COVID-19 crisis will not immediately translate into potentially inadequate structural adjustment requirements according to the matrix. This is because the SGP's general escape clause will remain in place at least in 2020. However, the difficulty with assessing the extent to which the COVID-19 shock and the associated exceptionally high degree of uncertainty translates into lasting scars and thus lower potential output in the long run will prevail. This will undermine the meaningfulness of granular adjustment requirements over the coming years.

An additional factor undermining the quality of the 'standard' methods of gauging potential output over the medium term relates to the difficulties of assessing the impact of the EU's unprecedented measures in support of the recovery from the COVID-19 shock. On 17-21 July 2020, the European Council agreed on an EU recovery package, called Next Generation EU, worth up to €750 billion. For the euro area as a whole, this package will provide sizeable financial support of almost 5% of GDP over the period 2021-26 (when measured in 2018 prices). At the heart of

6 See for details a forthcoming article in ECB's Economic Bulletin (2020).

7 It should be noted that the European Commission Spring 2020 Economic Forecast was released in May, i.e. at an earlier stage of the pandemic than the IMF's projections, which were released in July 2020. In an updated forecast released in July, the Commission pointed to a deeper than initially expected economic downturn following the pandemic. This forecast, however, does not entail updates of potential output and output gap estimates.

Figure 3
**The EU's Recovery and Resilience Fund: Implications for the euro area, 2021-26**
in % of GDP

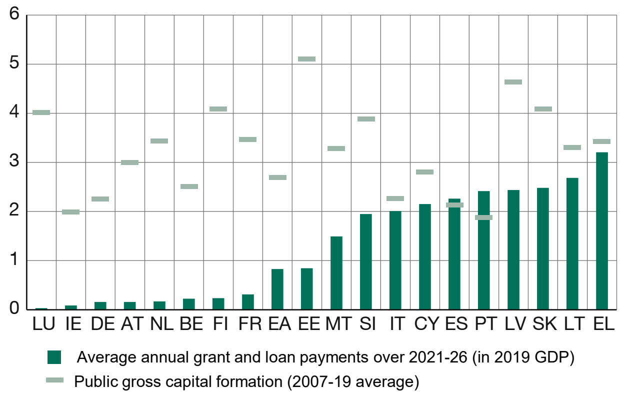

Sources: Council conclusions on Next Generation EU, European Commission Spring 2020 Economic Forecast, own calculations.

this recovery package is the EU Recovery and Resilience Facility (RRF), which makes up about 90% of total financial support. The RFF is intended to finance investment and structural reforms in the EU, with a major focus on supporting a transition towards greener and more digital economies. The setting in place of the RFF is associated with two imponderabilities for the measurement of the output gap. On the one hand, some countries are expected to receive a large amount of additional resources for government investment in a short period of time. For example, over the period 2021-26, Italy and Greece could receive each year financial support for additional investment of around 2% and 3% of GDP, respectively (see Figure 3). This implies that these countries could potentially double the public gross capital formation compared to the average of the past five years prior to the COVID-19 crisis. It will also be difficult to capture exactly how these additional resources will translate into higher potential output. This is all the more so as it is not yet clear to what extent the RRF will be used for *additional* investment and increase potential output accordingly, or rather replace existing capital spending plans. On the other hand, the intended shift towards more digital economies implies that a larger share of investment will be intangible. Such intangible investment is difficult to capture with traditional measures. Some literature has already pointed out that potential output – and negative output gaps accordingly - might be underestimated due to an increasing share of intangibles that are not captured by standard measures of potential output (Anderton et al., 2020). The difficulties of capturing such structural changes in an economy – and thus the size of the output gap – call for further caution with respect to resting fiscal policy guidance granularly on the unobservable output gap.

## Inconsistencies in the EU's fiscal framework

The SGP's preventive arm matrix is a reflection of a major inconsistency in the EU's fiscal framework. As it stands, the anchor of the EU's fiscal framework is countries' medium-term budgetary objective, which for signatories of the fiscal compact is defined as a nearly balanced budget in structural terms. If countries were to achieve such a balanced budget and maintain it over the long term, government debt-to-GDP ratios would theoretically converge to very low levels far below the Maastricht Treaty's threshold for government debt of 60% of GDP. This is in contradiction with the SGP's debt rule, which is supposed to steer countries with high government debt towards but not below the Maastricht threshold. Given that the SGP's debt rule has been de facto replaced in the past years by (broad) compliance with the preventive arm, the matrix requirements also pertain to countries with high government debt ratios. Consequently, the matrix can imply fiscal adjustment requirements that may be counterintuitive from the viewpoints of fiscal sustainability and stabilisation. Figure 4 illustrates information on the euro area countries in terms of different aspects. The colour coding reflects the European Commission's latest overall assessment of countries' debt sustainability, with risks rising with higher government debt-to-GDP ratios (European Commission, 2020a). The triangles refer to the structural adjustment requirements that countries would have needed to deliver in 2019, *ex post*, under the SGP's preventive arm.[8] The following are conclusions that can be drawn from Figure 4.

The matrix is a reflection of the fiscal framework's insufficient provision of room for stabilisation in countries with low government debt ratios. Notwithstanding low risks to government debt sustainability and sizeable fiscal space towards the Maastricht debt threshold, these countries need to deliver fiscal adjustment towards the SGP's anchor, i.e. the medium-term budgetary objective. For example, although Estonia posted a government debt-to-GDP ratio of below 10% of GDP in 2019, the matrix foresees a tightening of the structural balance of 0.5% of GDP. This was actually larger than the zero adjustment needed in some countries with very high debt ratios, which had achieved their medium-term budgetary objective, such as Cyprus and Greece. Overall, the constraints on fiscal policies for countries with low government debt limit the euro area's capability to provide needed fiscal support in times of low growth and inflation.

8 For this purpose, a few simplifying assumptions are made, see the note in Figure 4.

Figure 4

**Government debt-to-GDP ratios and output gap-based structural effort requirements, 2019**

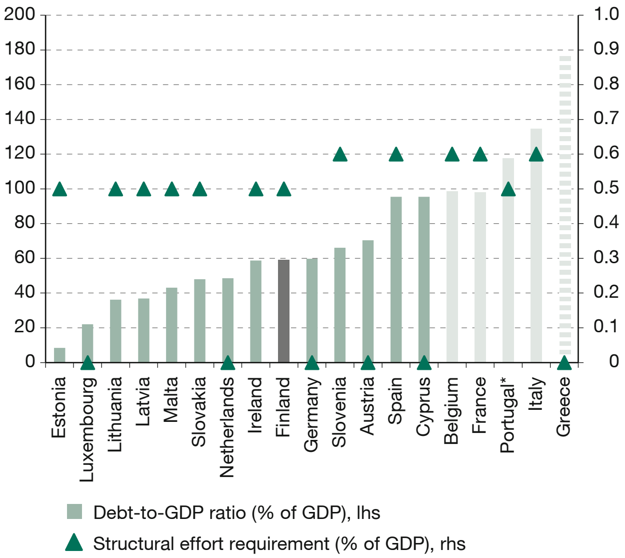

Notes: The structural effort requirements reflect the adjustment requirements that would have applied in retrospective had the matrix been applied, though with the following limitations: (i) the adjustment requirements may be lower for countries for which the *ex-ante* adjustment requirements relate to a lower output gap and associated smaller requirements; this is because the required adjustment cannot be larger *ex post* than what was required *ex ante*; ii) it may be higher for countries with closing and/or opening up output gaps. As regards the latter, the adjustment requirement of 1% has de facto never been applied. As regards the European Commission's debt sustainability assessment, Greece is coloured differently as it is not included in the Commission's assessment.

Sources: European Commission Spring 2020 Economic Forecast, European Commission Debt Sustainability Monitor 2019, own calculations.

The matrix is also a reflection of an insufficient differentiation among countries in terms of government debt ratios. As Figure 4 shows, a country such as Slovenia with government debt just above the Maastricht threshold would need to deliver the same structural adjustment as e.g. Italy, which recorded a debt ratio twice as high.

## Towards government debt as an anchor

The COVID-19 crisis and the exit from it will make estimates of the output gap even more difficult. This is likely to reduce the ownership of the associated fiscal adjustment requirements. Seeking the truly independent advice of, for example, national fiscal councils, may help in improving the reliability of output gap measures. It would, however, remain challenging to issue fully consistent policy advice.

The above analysis supports the calls for shifting the EU's fiscal framework towards government debt as an anchor. In principle, the SGP's debt rule is in place to ensure a gradual reduction of high debt towards the Maastricht reference value. As discussed in Hauptmeier and Kamps (2020), the existing debt rule of the SGP gives rise to a pro-cyclical bias which has hindered its implementation in the low-growth low-inflation environment. This calls for changes to the current specification of the rule in order to ensure that adjustment requirements towards the debt anchor appropriately reflect prevailing macroeconomic conditions so as to better balance the objectives of macroeconomic stabilisation and debt sustainability.

From a euro area perspective, it would be important for a reform of the fiscal framework to internalise the important interactions between monetary and fiscal policies as well as considerations related to the overall macroeconomic policy mix. First, the existing debt rule framework implies higher adjustment requirements when inflation is low. This is suboptimal from the viewpoint of the monetary policy authority given that fiscal support may be desirable in times of below-target inflation, especially when the effective lower bound has been reached. Second, the current framework is asymmetric in that it does not foresee the use of fiscal space when government debt is low and medium-term objectives have been overachieved. In this context, it has been argued that an effective coordination of the fiscal policy stance in the euro area is hindered by the inherent asymmetry of the rules.


Sebastian Hauptmeier, European Central Bank, Frankfurt am Main, Germany.

Nadine Leiner-Killinger, European Central Bank, Frankfurt am Main, Germany.


References

Anderton, R., V. Jarvis, V. Labhard, J. Morgan, F. Petroulakisans and L. Vivian (2020), Virtually everywhere? Digitalisation and the euro area and EU economies, ECB Occasional Paper, 244.
European Central Bank (2020), The impact of COVID-19 on potential output in the euro area, Economic Bulletin, forthcoming.
European Commission (2014), The Production Function Methodology for Calculating Potential Growth Rates & Output Gaps, Economic Papers, 535.
European Commission (2015), Making the best use of the flexibility within the existing rules of the Stability and Growth Pact, Communication from the Commission, COM(2015)12 final, https://eur-lex.europa.eu/legal-content/EN/TXT/?uri=celex%3A52015DC0012 (21 September 2020).
European Commission (2018), Communication from the Commission on the review of the flexibility under the Stability and Growth Pact, Commission staff working document, SWD(2018) 270 final, https://ec.europa.eu/info/sites/info/files/economy-finance/swd_2018_270_en.pdf (21 September 2020).
Hauptmeier, S. and C. Kamps (2020), Debt Rule Design in Theory and Practice: The SGP’s Debt Benchmark Revisited, ECB Working Paper, 2379.
Kamps C Leiner-Killinger N Taking stock of the EU’s fiscal rules over the past 20 years and options for reform Journal of Economics and Statistics 2019 239 5–6 861 894
Kamps, C., R. De Stefani, N. Leiner-Killinger, R. Rüffer and D. Sondermann (2014), The identification of fiscal and macroeconomic imbalances — synergies under the strengthened EU governance framework, ECB Occasional Paper, 157.
